# Comparative Effectiveness of Psychosocial Protective Factors for Prostate Cancer Survivorship ‐ A UK Biobank Study

**DOI:** 10.1002/pon.70258

**Published:** 2025-08-16

**Authors:** Yan Yu, Olivia Miu Yung Ngan, Varut Vardhanabhuti, Xin‐Yuan Guan, Fraide A. Ganotice

**Affiliations:** ^1^ Li Ka Shing Faculty of Medicine Bau Institute of Medical & Health Sciences Education The University of Hong Kong Hong Kong China; ^2^ Medical Ethics and Humanities Unit School of Clinical Medicine Li Ka Shing Faculty of Medicine The University of Hong Kong Hong Kong China; ^3^ Department of Diagnostic Radiology Li Ka Shing Faculty of Medicine The University of Hong Kong Hong Kong China; ^4^ Department of Clinical Oncology Li Ka Shing Faculty of Medicine The University of Hong Kong Hong Kong China

## Abstract

**Background:**

This study investigates psychosocial and lifestyle factors to improve survival outcomes in prostate cancer patients.

**Methods:**

From the UK Biobank cohort, 13,110 male prostate cancer subjects were analysed to examine the relationship between psychosocial and lifestyle factors and survival with a mean follow‐up of 14.2 years from recruitment.

**Results:**

Participation in sports club or gym (HR = 0.82, 95% CI 0.74–0.91, *p* < 0.005), religious groups (HR = 0.83, 95% CI 0.73–0.94, *p* < 0.005) and other group activity (HR = 0.87, 95% CI 0.78–0.97, *p* = 0.01) were associated with lower mortality risk in our analysis, after adjusting for age, deprivation and comorbidities, followed by. In contrast, neither the pub or social club nor the adult education class demonstrated a statistically significant survival benefit. A modest dose‐response relationship between the number of social activities engaged in and mortality risk reduction is observed. There were no differential benefits in alcohol intake, while smoking demonstrated a graded risk increase in mortality (HR = 1.74, 95% CI 1.51–2.0, *p* < 0.005 in current smokers; HR = 1.21, 95% CI 1.1–1.32, *p* < 0.005 in previous smokers) compared to never smokers. Having close and frequent confidants (HR = 0.83, 95% CI 0.75–0.92, *p* < 0.005 for daily) also confers benefits to survival.

**Conclusions:**

This study demonstrates that participation in sports club or gym, engaging in religious groups and other group activities, forming good health habits such as smoking cessation, and having people to confide in regularly is associated with reduced mortality risk in prostate cancer patients. These findings highlight the importance of integrating psychosocial resilience, health behaviour optimisation, and spiritual engagement into survivorship care. The hierarchical risk reduction profile supports prioritising interventions targeting modifiable health behaviours, spiritual/social support, and group activities. While religious participation is associated with notable survival benefits, this study recognises the complex interplay of cultural, social, and personal factors influencing engagement in such activities. These findings advocate for stratified survivorship care models prioritising engagement modalities with dual physiological, psychosocial, and spiritual benefits.

## 1 Background

Prostate cancer is the second most common cancer in men and the fifth leading cause of death globally [1] but the quantifiable impact of psychosocial factors on survival remains understudied. In 2018, approximately 1.3 million new cases and 360,000 fatalities were reported worldwide. Diagnosis typically begins with prostate‐specific antigen (PSA) screening; elevated levels prompt imaging (e.g., MRI) and biopsy [2]. Treatment depends on the stage of disease and varies from active surveillance, chemotherapy, radiation therapy, hormonal therapy, surgery, and cryotherapy [3], with 5‐year, 10‐year, and 15‐year survival rates nearing 100%, 98%, and 94%, respectively [4]. Prolonged survival necessitates identifying ways to minimise the risk of cancer progression, particularly as patients navigate treatment side effects, especially after prostatectomy, such as urinary incontinence, erectile dysfunction [5], changes in intimate relationships and sexuality [6].

Emerging evidence underscores modifiable factors [4], such as diet and lifestyle, in mitigating prostate cancer progression and improving survival, some of which diverge from those that are beneficial to healthy men, as cancer poses unique physiological and psychological challenges [7]. Physical exercise and weight maintenance are frequently complicated by treatment‐related fatigue [8] and psychological distress [9], highlighting the need to find ways to effectively adapt to the ‘new normal’ [10].

While psychological distress, including depression and anxiety, is widely recognised to negatively impact treatment adherence and outlook, bed confinement, and mortality risk across cancer populations [11, 12, 13], its unique manifestations in prostate cancer patients remain understudied and undertreated [9, 14]. Prostate cancer patients face distinct psychosocial burdens, including loss of personal control from functional decline (24% on androgen deprivation therapy report impaired Activities of Daily Living; 22% experiencing falls [15]) and identity disruptions from threats to masculinity, sexuality, and autonomy [16], correlating with poorer prognosis, particularly in metastatic disease [13]. This distress is multifaceted, encompassing eroded self‐confidence, diminished social connectivity, and existential loss of control over one's future [16], underscoring the need for targeted psychosocial interventions.

Religion is another survival determinant [17], with the majority of over 3000 quantitative studies up to 2008 linking increased survival rates with religiosity and spirituality [18]. For a few that did not, increased social support and protection from depression among the religious were noted [19], though most focussed on healthy individuals. In prostate cancer cohorts, the sample sizes were often small, but spirituality was found to play a significant role in coping and adjustment. One study [20] with 254 prostate cancer patients in the U.S. reported higher levels of daily spiritual experiences compared to the general population. Another African‐American study (*n* = 14) found that patients desired the integration of spirituality into their medical care [21]. Larger longitudinal studies are warranted. These findings highlight the need for holistic cancer care for the whole person, addressing emotional, spiritual, social [22], and lifestyle factors [23] through multidisciplinary collaboration [24].

This study analyses psychosocial engagements, religious participation, behavioural factors and mortality in the UK Biobank prostate cancer cohort, selected for (1) large sample size, rare in existing literature with statistical power allowing for the quantification of intervention efficacies; (2) long follow‐up and (3) further exploration due to conflicting evidence on religiosity and mortality in males [25], and social networks and prostate cancer survival [26]. We hypothesise that specific lifestyle modifications and targeted participation in certain psychosocial and faith‐based interventions correlate with differential survival among prostate cancer patients, measurable through robust statistical frameworks.

## 2 Methods

### 2.1 Study Design and Population

**FIGURE 1:**
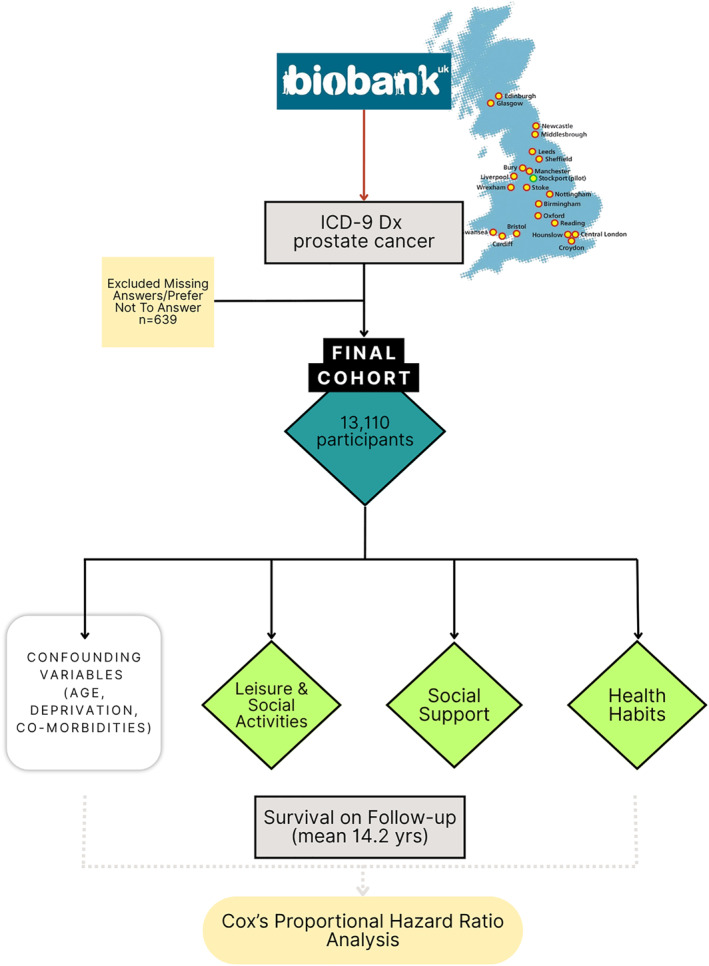
Overview of included data cohorts from the UK Biobank population and statistical analysis workflow.

The UK Biobank data, with the initial enroled participants between March 2006 and October 2010 aged between 40 and 69, was used for this study. Figure 1 describes the patient selection procedure and outcome variables being examined.

The UK North West Multi‐Centre Research Ethics Committee granted ethical approval for the UK Biobank study under reference 11/NW/0382. All participants gave informed written consent. The study was approved by the authors' local ethics board (UW‐20814) at the University of Hong Kong (using the UK Biobank Resource under Application Number 78730).

### 2.2 Inclusion/Exclusion Criteria

From the initial cohort of 501,314, we identified 13,739 men with a previous diagnosis of prostate cancer, defined by the International Classification of Diseases 10th Revision (ICD‐10) (Please refer to Supplementary Section A, for details). These cases were located through linked cancer registries in England, Scotland and Wales. We then excluded those who provided blank, ‘prefer not to answer’, or ‘do not know’ responses to any of the outcome or exposure variables (*n* = 639). The final cohort size 'is 13,110.

### 2.3 Outcome Variables

2204 all‐cause mortality events were observed during the follow‐up period. Survival time was computed as the duration (in days) from the initial cancer diagnosis date to either the mortality event or the last censor date (01 April 2024), whichever occurred first. The mean follow‐up spanned 14.2 years.

### 2.4 Exposure Variables

Our research explored social support data (Data field 100,061) and lifestyle and environment data (Data field 100,050).

#### 2.4.1 Social Support Data

The UK Biobank organises the social support data into three categories: ‘frequency of friend/family visits’ (including the pilot study), ‘leisure/social activities’, and ‘able to confide’.

Weekly leisure/social engagements were surveyed via structured questionnaire (Data Field 6160): ‘Which of the following do you attend once a week or more often?’ Responses were selected from a predefined categorical list: ‘Sports club or gym’, ‘Pub or social club’, ‘Religious group’, ‘Adult educational class’, ‘Other group activity’, ‘None of the above’ or ‘Prefer not to answer’. Multiple selections were permitted. A subset analysis was conducted excluding participants with concurrent activities to mitigate crossover effects.

Social support frequency was evaluated using ‘How often are you able to confide in someone close to you?’ (Data Field 2110). A single response was selected from ‘Almost daily’, ‘2–4 times a week’, ‘About once a week’, ‘About once a month’, ‘Once every few months’, ‘Never or almost never’, ‘Do not know’ or ‘Prefer not to answer’. Responses were categorised into ascending frequency groups of 0–3, with Group 0 as baseline (see Supporting Information S1: Table 1).

#### 2.4.2 Lifestyle and Environment Data

Health‐related behaviours (tobacco use Data Field 20,116 and alcohol consumption Data Field 20,117) were self‐reported. Participants selected status as ‘Current’, ‘Previous’, ‘Never’, or ‘Prefer not to answer’.

### 2.5 Confounding Variables

Age was included as a confounder due to its correlation with prostate cancer incidence and mortality [1]. Townsend deprivation index, a validated area‐level material deprivation metric [27], calculated based on four census variables, namely, car ownership, overcrowded households, owner‐occupation, and persons unemployed, was also included.

Cancer patients with comorbidities are known to have worse survival outcomes [28]. Chronic diseases were included as a confounder in the analysis of leisure/social activities and mortality. To determine the occurrence of chronic diseases, data sources included ICD‐10 codes, self‐reported data collected through interviews with healthcare professionals, records from general practitioners, and national death registries. For detailed information on the UK Biobank data field and ICD‐10 codes, please refer to Supplementary Material B.

Self‐reported ethnicity (unadjusted) demonstrates a limited discriminatory effect in UK health studies due to homogeneous British identification irrespective of ancestral heritage [29]. Gender was unadjusted due to male‐only cohort.

### 2.6 Statistical Analysis

Statistical analyses were conducted using Python (including NumPy, Pandas, Statistics, and Lifelines packages) to evaluate mortality risk associations with lifestyle and environment profiles (alcohol and smoking status), leisure/social engagement patterns, and social support adequacy. Survival analyses were conducted via Cox's Proportional Hazard Ratio (HR), which is one of the most widely used tools [30] for modelling the relationship of covariates to survival or censored outcomes. A *p*‐value of < 0.05 was considered statistically significant.

## 3 Results

Out of the 13,110 male subjects included in the study, the average age is 61.7 years, and the standard deviation is 5.7. A total of 11,937 individuals (91.1%) self‐identified as British. In terms of leisure and social activities, the total count of leisure and social activities is 13,407, as each participant can take up multiple activities. For detailed demographics, please refer to Table 1.

**TABLE 1:** Demographic overview of cohort population.

Age at recruit	*n* (%)
40–49	547 (4.2%)
50–59	3170 (24.2%)
60–69	9393 (71.6%)
Ethnicity	
British	11,937 (91.1%)
Irish	339 (2.6%)
Any other white	273 (2.1%)
Caribbean	183 (1.4%)
African	105 (0.8%)
All others	273 (2.0%)
Leisure/Social activities^a^	
Pub/social club	4571 (34.1%)
Sports club/gym	3780 (28.2%)
Other group activity	2530 (18.9%)
Religious group	1873 (14.0%)
Adult education	653 (4.9%)
Social support frequency	
Never/almost never^b^	3172 (24.2%)
∼Once a month	600 (4.6%)
1–4 times/week	2058 (15.7%)
Almost daily	7280 (55.5%)
Lifestyle/Environment	
Smoking status	
Never smokers	6401 (48.8%)
Ex‐smokers	5576 (42.5%)
Current smokers	1133 (8.6%)
Alcohol consumption	
Never drinkers	322 (2.5%)
Ex‐drinkers	381 (2.9%)
Current drinkers	12,407 (94.6%)

^a^ Total count of leisure activities reflects the sum of all activities reported by participants, including those engaging in multiple activities.

^b^ Includes ‘Once every few months'..

### 3.1 Univariate Analysis

#### 3.1.1 Confounding Variables

The results show that the variables considered to be potential confounders a priori were found to be significantly associated with mortality in univariate models (see Table 2).

**TABLE 2:** Univariable analysis demonstrating the impact of confounding factors on mortality.

	Hazard ratio	95% CI	*p*‐value
Age	1.07**	1.06–1.08	< 0.005
Townsend deprivation index	1.05**	1.04–1.07	< 0.005
Comorbidities at recruitment	1.50**	1.38–1.63	< 0.005

*Note:* Significance levels *p < 0.05, **p < 0.005.

#### 3.1.2 Exposure Variables

Univariate analysis was also conducted to assess the relationship between each exposure variables and mortality individually via Cox models. The results are summarised in Table 3.

**TABLE 3:** Univariable analysis demonstrating the impact of exposure factors on mortality.

	Hazard ratio	95% CI	*p*‐value
Religious group	0.79**	0.70–0.90	< 0.005
Sports club or gym	0.79**	0.71–0.87	< 0.005
Pub or social club	1.03	0.95–1.13	0.45
Other group activities	0.85**	0.76–0.95	< 0.005
Adult education class	0.84	0.68–1.03	0.09
Smoking (previous)	1.23**	1.12–1.34	< 0.005
Smoking (current)	1.85**	1.61–2.12	< 0.005
Alcohol (previous)	1.60	1.17–2.19	< 0.005
Alcohol (current)	1.00	0.78–1.29	0.98
Confide (monthly)	0.87	0.71–1.08	0.21
Confide (1–4 times/week)	0.81**	0.71–0.93	< 0.005
Confide (daily)	0.81**	0.73–0.89	< 0.005

*Note:* Age, deprivation and comorbidities were included as independent variables (covariates) in this model. Significance levels *p < 0.05, **p < 0.005.

### 3.2 Multivariable Analysis

Multivariable analysis was then conducted, where all valid variables and confounders were included in the Cox model at once. The results are presented in Table 4.

**TABLE 4:** Multivariable analysis demonstrating the impact of exposure factors on mortality.

	Hazard ratio	95% CI	*p*‐value
Age	1.07**	(1.06–1.08)	< 0.005
Deprivation	1.03**	(1.02–1.05)	< 0.005
Comorbidities	1.34**	(1.23–1.46)	< 0.005
Religious group	0.83**	(0.73–0.94)	< 0.005
Sports club or gym	0.82**	(0.74–0.91)	< 0.005
Pub or social club	1.03	(0.94–1.13)	0.49
Adult education class	0.87	(0.71–1.06)	0.17
Other group activity	0.87*	(0.78–0.97)	0.01
Alcohol (previous)	1.44*	(1.05–1.98)	0.02
Alcohol (current)	0.89	(0.69–1.15)	0.38
Smoking (previous)	1.21**	(1.1–1.32)	< 0.005
Smoking (current)	1.74**	(1.51–2)	< 0.005
Confide (monthly)	0.91	(0.74–1.12)	0.38
Confide (1–4 times/week)	0.84*	(0.73–0.96)	0.01
Confide (daily)	0.83**	(0.75–0.92)	< 0.005

*Note:* Significance levels *p < 0.05, **p < 0.005.

#### 3.2.1 Leisure and Social Activities and Mortality

The results showed statistically significant survival benefit for participation in religious group, sports club or gym, and other group activity (see Table 4). Sports club or gym is associated with the highest protection with a 18% reduction in mortality risk, followed by religious group (17%) and other group activity (13%). However, the relationship between survival and participating in pub or social club and adult education class were not statistically significant.

##### 3.2.1.1 Subset Analysis for Participation in Single Activity

To mitigate the crossover effects between different activities, a subset analysis of participants who only joined one activity, the cohort size was reduced by 25.7% to 10,210, and the results are presented in Table 5. The overall trend remains similar to that of the overall cohort. Religious participation, sports club or gym and other group activity for the subset offers 4%, 10% and 5% more protective effects, respectively.

**TABLE 5:** Leisure and social activities and mortality (confounding variables adjusted) for subset of those who participated in only one activity.

	Hazard ratio (subset)	Hazard ratio (all)	95% CI (subset)	*p*‐value
Religious group	0.79*	0.80**	0.65–0.96	0.02
Sports club or gym	0.72**	0.78**	0.62–0.83	< 0.005
Pub or social club	1.04	1.01	0.93–1.17	0.47
Other group activity	0.82*	0.84*	0.70–0.97	0.02
Adult education class	0.89	0.86	0.62–1.27	0.52

*Note:* Age, deprivation and comorbidities were included as independent variables (covariates) in this model. Significance levels *p < 0.05, **p < 0.005.

##### 3.2.1.2 Does‐Response in Social Interactions

To understand the cumulative effects of different social integrations, Hazard Ratio was calculated for participation in 1, 2, 3, 4 or 5 activities, and the results are presented in Table 6. As a subject participates in more activities, there is a trend towards a reduction in the Hazard Ratio, suggesting dose‐response. However, results are not statistically significant for the group with four activities, likely owing to the small number of subjects. Only 3 subjects participated in all five activities, the results of this group are not shown here.

**TABLE 6:** Leisure and social activities and mortality (according to number of activities participated).

	Hazard ratio	Participants (n)	95% CI	*p*‐value
One activity	0.88*	6010	0.80–0.97	0.01
Two activities	0.74**	2835	0.66–0.84	< 0.005
Three activities	0.67**	511	0.52–0.85	< 0.005
Four activities	0.67	46	0.32–1.42	0.3

*Note:* Age, deprivation and comorbidities were included as independent variables (covariates) in this model. Significance levels *p < 0.05, **p < 0.005.

#### 3.2.2 Social Support and Mortality

The results demonstrated a significant positive association between higher confiding frequency and survival outcomes (see Table 4). Compared to the baseline of never or almost never confiders, confiding in others daily or 1–4 times per week are associated with similar levels of mortality risk reduction, of 17% and 16% respectively. The relationship between survival and confiding monthly was not statistically significant.

#### 3.2.3 Lifestyle/Environment and Mortality

A protective effect for non‐smoking and smoking cessation was found (see Table 4). Compared to the baseline of never smokers, current smokers see their mortality risk increase by 74%, whereas previous smokers have a lower mortality risk increase of 21%.

The relationship between mortality risk and alcohol drinker status remains mixed, producing a statistically significant adverse relationship for alcohol cessation and a non‐significant association for current drinkers.

## 4 Discussions

Engagement in sports club or gym, religious groups, other group activity, and having close confidants all appear to improve survival among prostate cancer patients, independent of age, deprivation, comorbidity, smoking, and alcohol intake. In contrast, increasing age, greater social deprivation, more comorbidities, and smoking (particularly current smoking) are associated with decreased survival. Pub or social club attendance and adult education classes were not significantly related to survival in our analysis.

We are one of the first teams to use biobank data to examine modifiable psychosocial and behavioural factors in cancer survival analysis, while other studies tend to focus more on lifestyle factors [31, 32, 33]. For example, a meta‐analysis combining 16 studies that totalled 1.9 million participants found that adopting a healthy lifestyle is associated with a substantial risk reduction in cancer morbidity and mortality [34]. In our study, other than confirming the positive effects of physical activities and smoking cessation from lifestyle studies on cancer survival, we also found that modifiable psychosocial and behavioural factors such as participation in religious groups and other group activities, having people to confide with at least weekly are associated with improve survival in prostate cancer patients. While some prior studies have established the prognostic relevance of these variables in heterogeneous cancer populations [35, 36, 37], certain prospective investigations and systematic reviews did not find an association [38] or resulted in inconsistent findings across studies [39]. Our study represents the first demonstration to show the benefits in a large cohort [40] over a relatively long follow‐up period for a single disease. However, since it was not possible to obtain data on the racial/ethnic backgrounds of the participants, we were unable to investigate racial/ethnic differences in this study. Further analysis on the effect of psychosocial variables in Asian [41]or African [42] cohorts on mortality would be beneficial.

A key contribution of this analysis lies in strengthening empirical evidence for the association of religious participation and mortality reduction [43], and confirms its protective effect in the male population [19, 44]. For example, a number of research papers found a relationship between religiosity and mortality reduction that was significant for females but not males [25]. This study provides a focussed analysis, based on UK Biobank data, showing that religious participation is associated with a protective effect on mortality risk for prostate cancer patients and survivors, which had previous been under‐researched.

Another important contribution of this study lies in revealing differential survival benefits across psychosocial modalities in prostate cancer patients. While prior studies have acknowledged variability in psychosocial determinants of survival [45], this analysis provides quantification of these disparities, enabled by longitudinal cohort data. Notably, non‐targeted social interactions (pub/social club attendance) and adult education classes showed no significant survival benefits. In the case of pub/social club, the result potentially reflects concurrent health‐risk behaviours in the setting (e.g., smoking, excessive alcohol consumption) [46]. While religious participation is associated with reduced mortality, further investigation is necessary to fully account for potential confounding factors. These findings advocate for stratified survivorship care paradigms prioritising engagement modalities with dual physiological and psychosocial benefits.

In the area of health practices, the mixed results from alcohol consumption status merit cautious interpretation, given the cohort's high baseline prevalence of current drinkers (94.3%) and absence of granular exposure metrics within the UK Biobank. Debate persists regarding the potential health benefits of moderate consumption of certain types of alcohol [47]. Further investigations, including type‐frequency‐volume assessments, are critical to yield more fruitful survival analysis on this topic.

Our analysis also revealed a nonlinear dose‐response relationship between confiding frequency and mortality risk in prostate cancer survivorship. While a threshold effect was observed, with ≥ weekly confiding associated with a ≥ 16% mortality risk reduction, beyond this threshold, marginal gains diminished. This seems to suggest that psychosocial protective mechanisms saturate at moderate interaction frequencies in prostate cancer patients. Conversely, infrequent confiding (monthly or less) showed no statistically significant survival benefit, potentially reflecting insufficient activation of protective effects. For clinical translation, this supports ensuring consistent weekly confiding opportunities while preventing high‐frequency mandates that over‐burdens resource constraints.

This investigation has several methodological constraints requiring acknowledgement. First, the cancer type–specific focus on prostate malignancies limits generalisability; comparative cross‐cancer analyses are warranted to assess the universality of observed mortality associations. Second, it would be beneficial to also consider cancer stages, grades and treatments as these factors affect mortality. Unfortunately, these data were not available in the UK Biobank data. Thirdly, while we adjusted for age, deprivation and comorbidities, there is an argument for the (1) persistent residual confounding and (2) overadjustment bias. Residual confounding factors may persist due to unmeasured socioeconomic determinants and granular health metrics. Future studies should incorporate adjustments for these covariates to enhance causal inference. Addressing these limitations through multinational cohorts with multidimensional exposure assessments remains critical for advancing survivorship frameworks. Both the social deprivation score and co‐morbidities in the survival model can also lead to overadjustment, because co‐morbidities may be part of the causal pathway from social deprivation to survival. This can underestimate the total effect of social deprivation on survival. Lastly, this study did not examine potential geo‐cultural specificity in socioreligious survival associations, a critical limitation given emerging evidence of context‐dependent effects. While prior research has established robust lifestyle‐survival association in the US, UK and China [31], as well as religion‐health linkages in U.S. cohorts [48], it may not be applicable to the rest of the world. Our UK‐based findings extend evidence to a secularised European context. However, cross‐cultural generalisability remains constrained by the absence of data from regions with different socioreligious ecosystems, particularly Asian and sub‐Saharan African contexts where spiritual practices are often interwoven with traditional healing modalities. Such investigations could clarify whether religiosity's survival benefits represent universal or culturally bounded phenomena that weaken in secularised populations [49], thereby informing context‐specific survivorship care strategies.

## Author Contributions

Initial Project Conception: Y.Y., V.V., O.N., F.A.G. Data Curation: Y.Y., V.V. Data Analysis: Y.Y., V.V. Initial Manuscript Draft: Y.Y. Manuscript Editing: Y.Y., O.N., V.V., X.G., F.A.G., Final Manuscript Review: Y.Y., O.N., V.V., X.G., F.A.G.

## Conflicts of Interest

The authors declare no conflicts of interest.

## Supporting information


Supporting Information S1

## Data Availability

The data used in this study is from the UK Biobank. Researchers are able to apply for access for the datasets to conduct their own independent research and validation. The UK Biobank does not allow for sharing of its data.
