# Supplementary material for: Comparative Effectiveness of Psychosocial Protective Factors for Prostate Cancer Survivorship ‐ A UK Biobank Study
Source: Psychooncology. 2025 Aug 16;34(8):e70258. doi: 10.1002/pon.70258 (PMC12357647; doi:10.1002/pon.70258)
Supplement: Supplementary file 1 — Supporting Information S1 [file PON-34-e70258-s001.docx]

**Supplementary Materials**

| **Frequency** | **Assigned Group** | **Code** |
| --- | --- | --- |
| Never or almost never | Never | 0 |
| Once every few months | Once every few months | 0 |
| About once a month | Monthly | 1 |
| 2-4 times a week | 1-4 times a week | 2 |
| About once a week | 1-4 times a week | 2 |
| Almost daily | Daily | 3 |

**Supplementary Table 1 Confiding frequency grouping for analysis (confounding variables adjusted)**


Supplementary Material A: Coding for Prostate Cancers

Prostate cancer cohorts were filtered using these ICD 9 coding

C61 Malignant neoplasm of prostate

D07.5 Prostate

D40.0 Prostate

This resulted in 13740 subjects.

One subject ID 2572792 identified as female in gender cohort. It is not clear the reason for this, though it may be that the subject was born as male. This subject was removed to avoid ambiguity, so final number is 13739.

Supplementary Material Section B:

International Classification of Diseases (ICD) for the chronic diseases

Codes of International Classification of Diseases (ICD) were used to adjudicate HTN cases (ICD-10: I10, I11, I12, I13, I16), ASCVD cases (ICD-10: I25), CVD cases (ICD-10: I60, I61, I63, I64), ND cases (ICD-10: A81, F00, F01, F02, F03, F05, F10, G30, G31, I67, G20), MetS cases (ICD-10: E70, E71, E72, E73, E74, E75, E76, E77, E78, E79, E80, E83, E84, E85, E86, E87, E88, E89, E90) and T2DM cases (ICD-10: E11).

The outcomes and diseases include major adverse cardiac event, type 2 diabetes, liver disease, renal disease, atrial fibrillation, heart failure, ischaemic heart disease, venous thrombosis, cerebral stroke, abdominal aortic aneurysm, peripheral arterial disease, cataracts, glaucoma, hypertension, atherosclerotic cardiovascular disease, asthma, chronic obstructive pulmonary disease, Parkinson, dementia and metabolic syndrome.
